# 

**Published:** 1896-03

**Authors:** 


MARCH, 1896.

*Vol. XIV.* *No.* **51.**

### THE BRISTOL MEDICO-CHIRURGICAL JOURNAL

**A Quarterly Journal of the Medical Sciences for the West of England and South Wales**

*PUBLISHED UNDER THE AUSPICES OF*
*THE BRISTOL MEDICO-CHIRURGICAL SOCIETY.*

"Scire est nescire, nisi id me

Scire alius sciret."

LIBRARY OF CONGRESS
34777
DEC 1 1897
SMITHSONIAN DEPOSIT.

*Editor:* R. SHINGLETON SMITH, M.D., B.Sc.
WITH WHOM ARE ASSOCIATED
J. MICHELL CLARKE, M.A., M.D.
F. R. CROSS, M.B. AND W. H. HARSANT.
*Assistant-Editor:* L. M. GRIFFITHS.
*Editorial Secretary:* B. M. H. ROGERS, B.A., M.D., B.Ch.

BRISTOL: J. W. ARROWSMITH;
London: J. & A. Churchill, 7 Great Marlborough Street.

*Price One Shilling and Sixpence.*

